# Fatal Case of Chronic Jamestown Canyon Virus Encephalitis Diagnosed by Metagenomic Sequencing in Patient Receiving Rituximab

**DOI:** 10.3201/eid2701.203448

**Published:** 2021-01

**Authors:** Isaac H. Solomon, Vijay S. Ganesh, Guixia Yu, Xian Ding Deng, Michael R. Wilson, Steve Miller, Tracey A. Milligan, Shibani S. Mukerji, Abigail Mathewson, Justin Linxweiler, Darlene Morse, Jana M. Ritter, J. Erin Staples, Holly Hughes, Carolyn V. Gould, Pardis C. Sabeti, Charles Y. Chiu, Anne Piantadosi

**Affiliations:** Brigham and Women’s Hospital, Boston, Massachusetts, USA (I.H. Solomon, V.S. Ganesh, T.A. Milligan);; Harvard Medical School, Boston (I.H. Solomon, V.S. Ganesh, T.A. Milligan, S.S. Mukerji, A. Piantadosi);; Broad Institute, Cambridge, Massachusetts, USA (V.S. Ganesh, P.C. Sabeti, A. Piantadosi);; University of California–San Francisco, San Francisco, California, USA (G. Yu, X.D. Deng, M.R. Wilson, S. Miller, C.Y. Chiu);; Massachusetts General Hospital, Boston (S.S. Mukerji, A. Piantadosi);; New Hampshire Division of Public Health Services, Concord, New Hampshire, USA (A. Mathewson, J. Linxweiler, D. Morse);; Centers for Disease Control and Prevention, Atlanta, Georgia, USA (J.M. Ritter);; Centers for Disease Control and Prevention, Fort Collins, Colorado, USA (J.E. Staples, H. Hughes, C.V. Gould);; Harvard University, Cambridge (P.C. Sabeti);; Harvard T.H. Chan School of Public Health, Boston (P.C. Sabeti);; Howard Hughes Medical Institute, Chevy Chase, Maryland, USA (P.C. Sabeti)

**Keywords:** orthobunyavirus, arbovirus, Jamestown Canyon virus, encephalitis, rituximab, metagenomic next-generation sequencing, viruses, vector-borne infections, monoclonal antibody

## Abstract

A 56-year-old man receiving rituximab who had months of neurologic symptoms was found to have Jamestown Canyon virus in cerebrospinal fluid by clinical metagenomic sequencing. The patient died, and postmortem examination revealed extensive neuropathologic abnormalities. Deep sequencing enabled detailed characterization of viral genomes from the cerebrospinal fluid, cerebellum, and cerebral cortex.

Jamestown Canyon orthobunyavirus (JCV) is a negative-sense RNA virus in the California serogroup. Its tripartite genome comprises small (nucleocapsid), medium (glycoprotein), and large (polymerase) segments. JCV is distributed throughout the United States and Canada and has been isolated from multiple mammals and mosquitoes (*1*,*2*). Most infections occur in adults, during the summer, and are asymptomatic, but manifestations can include fever and acute meningoencephalitis (*2*). Cerebrospinal fluid (CSF) typically shows a lymphocytic pleocytosis with elevated protein and normal glucose. Diagnosis is made by detection of JCV IgM in serum or CSF and confirmed by plaque-reduction neutralization testing to rule out cross-reactivity with other California serogroup viruses (*3*). Detection of viral RNA in human CSF has rarely been described, with viremia presumed to be of short duration, so reverse transcription PCR (RT-PCR) is not routinely used for diagnosis (*3*–*5*). No specific treatments are available, although intravenous ribavirin has been reported to improve seizures (*6*). Because of the limited number of cases described, the full range of findings associated with JCV infection is unknown. No fatal cases were reported to the Centers for Disease Control and Prevention (CDC) before 2017, and no autopsy reports have been published (*7*).

## The Case-Patient

**Figure 1:**
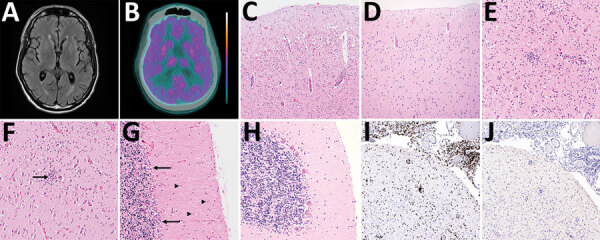
Brain imaging and autopsy findings in a case of chronic Jamestown Canyon virus (JCV) meningoencephalitis in a patient receiving rituximab, Boston, Massachusetts, USA. A) Brain magnetic resonance imaging T2-weighted fluid-attenuated inversion recovery showed mild atrophy with secondary ventriculomegaly but was otherwise unremarkable. B) Brain positron emission tomography with 2-deoxy-2-[fluorine-18] fluoro-D-glucose integrated with computed tomography showed global hypometabolism. Color scale ranges from blue-green (hypometabolic) to orange-white (hypermetabolic). C, D) Hematoxylin and eosin stained section of cerebral cortex at low magnification shows loss of neurons and perivascular chronic inflammation (C), compared with a JCV-negative control with a normal complement of cortical neurons (D). E, F) Higher-power magnification of cerebral cortex (E) and hippocampus (F) show microgliosis, microglial nodules, and neuronophagia (arrow). G, H) Severe Purkinje cell loss, Bergmann gliois (arrows), and microgliosis (arrowheads) of the molecular layer are present in the cerebellum (G), compared with a JCV-negative control with normal complement of Purkinje cells (H). I, J) Immunohistochemistry shows abundant perivascular, parenchymal, and leptomeningeal CD3+ T cells (I) and is negative for B-cell lineage–specific activator protein positive B cells (J). Panels C, D, I, and J, original magnification ×100; panels E, F, G, and H, original magnification ×200.

A 56-year-old man from New England with a history of mantle cell lymphoma in remission, receiving maintenance rituximab since 2014, had fatigue, arthralgias, and weight loss in summer 2017. He was empirically treated for Lyme disease without improvement, had progressive insomnia and inattention, and was eventually admitted for workup of rapidly progressive dementia in April 2018. On examination, he had impaired arousal and attention (Montreal Cognitive Assessment score 6 of 30). Cranial nerve, tone, strength, sensory, and reflex examinations were normal. Gait was wide-based and slow without ataxia or parkinsonism. Magnetic resonance imaging of the brain showed mild ventriculomegaly attributed to atrophy but was otherwise unremarkable, without contrast enhancement, cortical diffusion restriction, mass lesions, hemorrhage, or infarction (Figure 1). Electroencephalography showed moderate bihemispheric slowing without epileptiform features. CSF from multiple lumbar punctures showed mild lymphocytic pleocytosis (0–22 leukocytes/μL, 83%–98% lymphocytes), elevated total protein (40–116 mg/dL; reference 10–44 mg/dL), and unremarkable glucose (65–78 mg/dL; reference 40–80 mg/dL) (Appendix Table 1). An extensive infectious, autoimmune, and neurodegenerative disease workup was normal (Appendix Table 2).

A CSF sample collected in April 2018 underwent clinical metagenomic next-generation sequencing (mNGS) testing at the University of California–San Francisco (*8*) and was positive for California encephalitis virus most closely matching JCV, with reads mapping to 2 of the 3 viral genome segments (Appendix Figure 1). Another CSF sample, obtained approximately 3 weeks later in May, was negative for JCV by RT-PCR performed by CDC’s Arboviral Diseases Branch (Division of Vector-Borne Diseases, National Center for Emerging and Zoonotic Infectious Diseases; Fort Collins, CO, USA); however, concurrent serum JCV RT-PCR was positive. Results of JCV IgM and neutralizing antibody testing were negative for CSF and blood from the samples obtained in May. Concurrent samples had 0% CD20+ circulating lymphocytes (reference 3%–20% lymphocytes), attributed to rituximab treatment, last administered in December 2017.

The patient was treated with intravenous immunoglobulin (total 2 g/kg), followed by a 2-week course of favipiravir, an experimental inhibitor of viral RNA polymerase, without improvement. His mental status deteriorated to a comatose state. He was transitioned to comfort care and died in June 2018, ≈1 year after suspected symptom onset.

At autopsy, the unfixed brain weighed 1,240 g and appeared grossly normal, with no masses, hemorrhage, infarctions, or herniation. Histologic abnormalities were most prominent in the cerebral cortex (particularly frontal and temporal lobes), cerebellum, and hippocampus; milder changes in basal ganglia, thalamus, and brainstem were observed, including severe loss of neurons, diffuse microgliosis with microglial nodules and neuronophagia, and perivascular and parenchymal chronic inflammation (Figure 1). Leptomeninges showed numerous chronic inflammatory cells. No viral inclusions were identified. There was no evidence of lymphoma. Immunohistochemical staining highlighted abundant perivascular, parenchymal, and leptomeningeal T cells with a complete lack of B cells. Formalin-fixed paraffin-embedded brain tissue was positive for JCV by RT-PCR (performed by CDC’s Arboviral Diagnostic and Reference Laboratory); results were negative for immunohistochemistry for flaviviruses and enteroviruses (performed by CDC’s Infectious Diseases Pathology Branch [Division of High-Consequence Pathogens and Pathology, National Center for Emerging and Zoonotic Infectious Diseases; Atlanta, GA, USA]).

**Figure 2:**
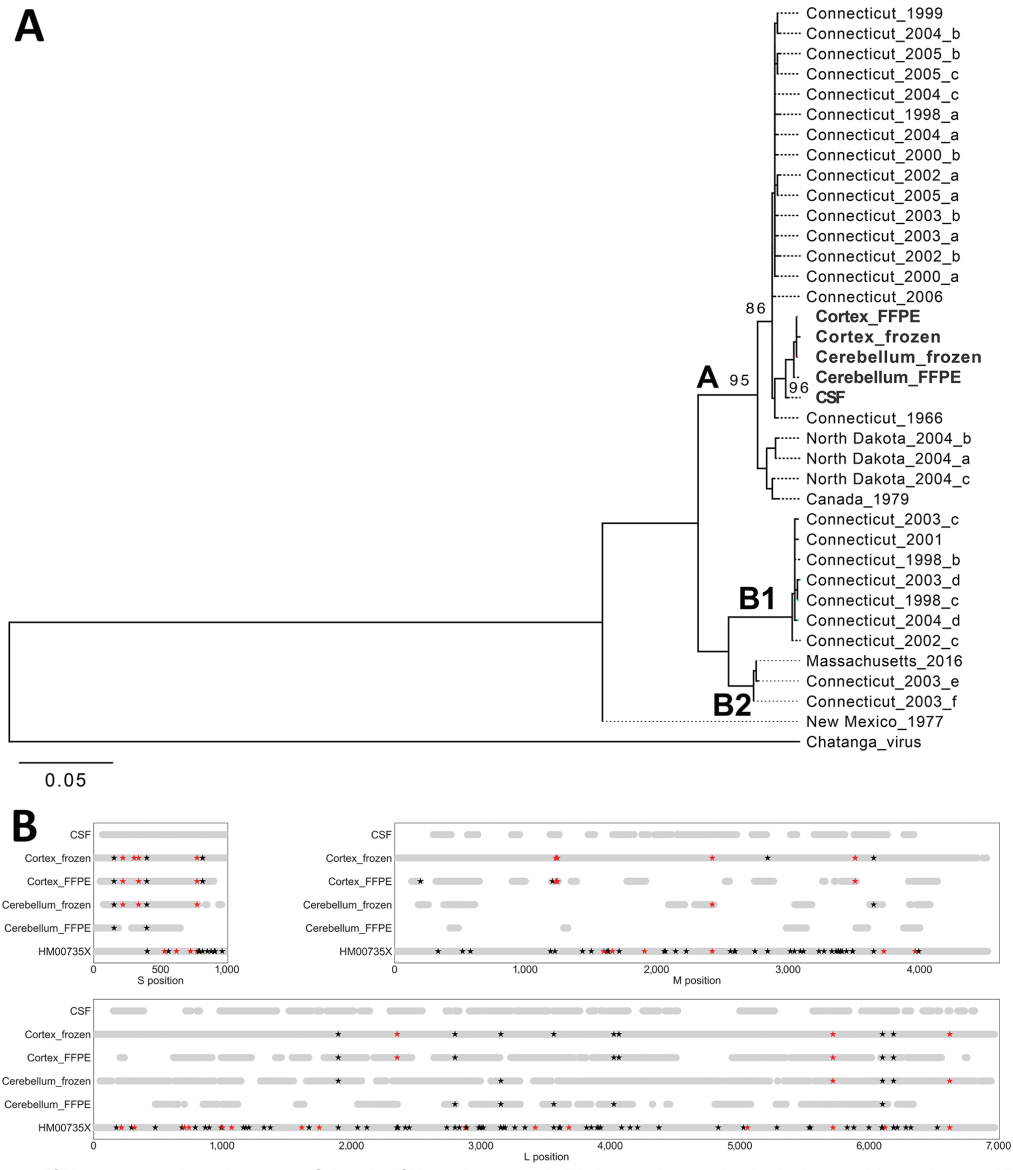
JCV genome analyses in a case of chronic JCV meningoencephalitis in a patient on rituximab, Boston, Massachusetts, USA. A) Maximum-likelihood phylogenetic tree of the coding region of the JCV small segment (nucleocapsid). Sequences from the patient (bold) were most closely related to a JCV strain isolated from Simsbury, Connecticut, USA (GenBank accession no. EF681842), with ≈70% bootstrap support. Clades A, B1, and B2 are as previously reported (*9*). B) Single-nucleotide polymorphisms (SNPs) observed between samples from patient in this study. The consensus genome derived from each sample was aligned to a mosquito-derived JCV sequence (GenBank accession nos. HM007356 [S segment], HM007357 [M segment], and HM007358 [L segment], all represented in the figure as HM00735X). For each sample in this study, the light gray bar indicates positions for which there was coverage of >3 reads. Using the sequence derived from CSF as the reference, positions with a SNP are marked with a star; black indicates a synonymous change, and red indicates a nonsynonymous change. Only high-confidence (confirmed) SNPs are shown in this figure; all SNPs observed are shown in Appendix Tables 3, 4. Sequence data is available under National Center for Biotechnology Information BioProject no. PRJNA662969 (GenBank accession nos. MW072986–MW073000). CSF, cerebrospinal fluid; FFPE, formalin-fixed, paraffin-embedded; JCV, Jamestown Canyon virus; L, large; M, medium; S, small.

Complete or near-complete JCV genomes were recovered from premortem CSF and postmortem cerebellum and cortex tissue (both frozen and formalin-fixed paraffin-embedded) (Table; Appendix Supplementary Methods, Figure 2). Phylogenetic analysis of the small (nucleocapsid) segment showed that sequences from this patient were most closely related to JCV from mosquitoes in Connecticut (Figure 2, panel A) (*9*). Comparison of JCV genomes between this patient’s CSF, cerebellum, and cortex revealed 27 high-confidence within-patient single-nucleotide polymorphisms (SNPs) (Figure 2, panel B; Appendix Tables 3, 4). For 13 SNPs, the variant present in CSF was different from that in cerebellum and cortex, suggesting evolution over time. For another 4 SNPs, the variant present in cerebellum was different from that in cortex, suggesting compartmentalization. The remaining 10 SNPs could represent either compartmentalization or evolution over time, because only 1 brain tissue (cerebellum or cortex) was sequenced to sufficient depth. Variability was greater in the small segment (nucleocapsid) and medium segment (glycoprotein) than the large segment (polymerase).

**Table:** Results of JCV sequencing across samples from an immunocompromised patient with encephalitis, Boston, Massachusetts, USA*

Specimen	Method	Total reads†	Unique JCV reads‡	% Genome assembled, by segment§				Mean depth, by genome segment§		
				Small	Medium	Large		Small	Medium	Large
CSF	mNGS, MSSPE	1,917,836,676	894	100	80	90		14.3	3.7	5.3
Cerebellum, frozen	mNGS, MSSPE	1,031,252,808	558	98	66	100		9.4	1.3	6.1
Cerebellum, FFPE	mNGS, hybrid capture	38,974,996	294	70	56	90		7.1	1.2	3.1
Cortex, frozen	mNGS, MSSPE	729,867,496	3,652	100	100	100		61.6	15.2	40.2
Cortex, FFPE	mNGS, hybrid capture	101,331,284	518	100	72	91		20.1	2.1	4.5

*Methods detailed in Appendix. CSF, cerebrospinal fluid; FFPE, formalin-fixed, paraffin-embedded; JCV, Jamestown Canyon virus; mNGS, metagenomic next-generation sequencing; MSSPE, metagenomic sequencing with spiked primer enrichment. †Total reads reflect the number of raw reads that were generated from each sample. ‡Unique JCV reads reflects removal of PCR duplicates and mapping to JCV reference sequences. §Small, 989 nt; Medium, 4,509 nt; Large, 6,960 nt.

## Conclusions

We describe an unusual fatal case of chronic JCV encephalitis in a patient who was being treated with rituximab. In contrast to this case, previously described patients with JCV have had acute illness, and JCV infection is rarely fatal (*7*,*10*). The neuropathologic findings in this patient, although nonspecific, are similar to those of a cerebellar biopsy from a patient with JCV encephalitis that showed severe loss of Purkinje and granule cells, diffuse microgliosis of the molecular layer, and leptomeningeal inflammation (*5*).

The lack of distinguishing clinical, radiographic, and pathologic features of JCV underscores the diagnostic utility of clinical mNGS (*8*). Attributable in part to low incidence and lack of commercially available targeted testing, JCV is often not considered a priori, especially in the setting of chronic progressive neurologic illness. As a further complication, standard clinical testing by serology can be negative in the setting of B-cell–depleting therapy; our patient had negative JCV serologic tests and lack of B lymphocytes by immunohistochemical staining. Similar phenomena have been reported in rituximab-treated patients with other arboviral infections (e.g., Cache Valley orthobunyavirus, Powassan virus, and West Nile virus) who lack detectable antibodies but remain viremic longer than immunocompetent patients, highlighting the importance of nucleic acid–based testing methods (*11*–*13*).

In addition to diagnosis, mNGS also provides valuable information about pathogen genomics. We report the unique assembly of a JCV genome from human clinical samples, an important advance in the study of JVC pathogenesis, virus evolution, and differences between the enzootic transmission cycle and human infection (*14*). The functional importance of the identified SNPs could not be evaluated from the genomic data alone; however, none were associated with alterations of potential N-linked glycosylation sites, cysteine bonds, or the conserved fusion domain (*15*). One SNP that arose between CSF and brain (small segment gA397G; aT109A) also varied between JCV strains with different neurovirulence in mice, although the functional importance is unknown (*4*).

Although treatment options for JCV infection are largely unexplored, response to antiviral drugs probably depends on initiating treatment early in the disease course and reaching therapeutic levels in the CSF before extensive neuronal loss. Thus, broad-spectrum molecular assays such as mNGS could potentially lead to earlier treatment with improved outcomes (*8*).

Appendix Additional information about a fatal case of chronic Jamestown Canyon virus encephalitis diagnosed by metagenomic sequencing in a patient receiving rituximab.
