# Supplementary material for: Fatal Case of Chronic Jamestown Canyon Virus Encephalitis Diagnosed by Metagenomic Sequencing in Patient Receiving Rituximab
Source: Emerg Infect Dis. 2021 Jan;27(1):238–42. doi: 10.3201/eid2701.203448 (PMC7774567; doi:10.3201/eid2701.203448)
Supplement: Appendix — Additional information about a fatal case of chronic Jamestown Canyon virus encephalitis diagnosed by metagenomic sequencing in a patient receiving rituximab. [file 20-3448-Techapp-s1.pdf]

Article DOI: https://doi.org/10.3201/eid2701.203448

Fatal Case of Chronic Jamestown Canyon Virus Encephalitis Diagnosed by Metagenomic Sequencing in a Patient Receiving Rituximab

Appendix

Supplementary Methods

This work was approved by the Partners Human Research Committee, the Institutional Review Board at Brigham and Women's Hospital. For viral genome recovery, mNGS was performed on CSF and post-mortem frozen brain tissue (cerebellum and cerebral cortex) using a metagenomic sequencing with spiked primer (MSSPE) approach employing random, arbovirus-specific, and JCV-specific spiked primers (*1*). In parallel, mNGS was performed on FFPE tissue from multiple brain sections (cerebellum, frontal lobe, insula, and temporal lobe) at the Broad Institute (Cambridge, MA), using published methods for mNGS and hybrid capture (*2*,*3*). Given the relatively low JCV recovery from some individual brain sections, reads from the frontal lobe, insula, and temporal lobe were merged (cerebral cortex), while reads from the cerebellum were analyzed independently.

From each sample, reads from human and known laboratory contaminants were removed, and reference-based assembly was performed using viral-ngs (*4*), with JCV references HM007356 (S segment), HM007357 (M segment), and HM007358 (L segment). For each sample, human reads were depleted, the remaining reads were mapped to the reference sequence (with a liberal minimum read depth of one), duplicates were removed, and the final reads were visually confirmed in Geneious (Biomatters, Inc., Auckland, New Zealand). The reported depth of coverage is based on unique JCV reads. The consensus JCV genomes from each sample were aligned in Geneious. To perform S segment phylogenetic analysis, genomes were aligned with all unique available S segment references on GenBank as well as the outgroup *Chatanga virus* (another California serogroup orthobunyavirus), sequences were trimmed to include only the

coding region, and maximum-likelihood trees were generated using PhyML (*5*). To identify SNPs, consensus JCV genomes were aligned as above and manually inspected for polymorphisms, using the patient CSF sample as a reference. SNPs were identified between CSF, cortex, and cerebellum samples (within-patient SNPs, Appendix Table 3), as well as between the patient samples and a reference sequence from mosquitoes (Appendix Table 4). As a conservative measure to help distinguish SNPs from sequencing artifacts, SNPs were considered high-confidence and "confirmed" only if detected with a depth of at least three unique JCV reads, and for within-patient SNPs in brain tissue, only if detected in both frozen and FFPE tissue. Sequence data is available under NCBI BioProject PRJNA662969 (GenBank accession nos. MW072986–MW073000).

References

1. Deng X, Achari A, Federman S, Yu G, Somasekar S, Bártolo I, et al. Metagenomic sequencing with spiked primer enrichment for viral diagnostics and genomic surveillance. Nat Microbiol. 2020;5:443–54. PubMed https://doi.org/10.1038/s41564-019-0637-9

2. Piantadosi A, Kanjilal S, Ganesh V, Khanna A, Hyle EP, Rosand J, et al. Rapid Detection of Powassan Virus in a Patient With Encephalitis by Metagenomic Sequencing. Clin Infect Dis. 2018;66:789–92. PubMed https://doi.org/10.1093/cid/cix792

3. Metsky HC, Siddle KJ, Gladden-Young A, Qu J, Yang DK, Brehio P, et al.; Viral Hemorrhagic Fever Consortium. Capturing sequence diversity in metagenomes with comprehensive and scalable probe design. Nat Biotechnol. 2019;37:160–8. PubMed https://doi.org/10.1038/s41587-018-0006-x

4. Park D, Tomkins-Tinch C, Jungreis I, Ye S, Metsky H, Lin M. broadinstitute/viral-ngs: v1.12.0. 2016. https://www.broadinstitute.org/viral-genomics/viral-genomics-analysis-apps

5. Guindon S, Dufayard JF, Lefort V, Anisimova M, Hordijk W, Gascuel O. New algorithms and methods to estimate maximum-likelihood phylogenies: assessing the performance of PhyML 3.0. Syst Biol. 2010;59:307–21. PubMed https://doi.org/10.1093/sysbio/syq010

Appendix Table 1. Cerebrospinal fluid test results by date

Test result	3/28/18	4/11/18	4/17/18	4/20/18	4/25/18	5/17/18	6/1/18	6/5/18
red blood cell (cells/µL)	57	163	12	7	153	2	10	4
leukocyte (cells/µL)	**14**	**11**	3	**20**	**12**	**8**	0	**22**
Differential	L 72%<br>M 7%<br>N 1%<br>A 20%	L 93%<br>M 5%<br>N 2%<br>A 0%	L 83%<br>M 1%<br>N 0%<br>A 16%	L 86%<br>M 1%<br>N 0%<br>A 10%	L 96%<br>M 3%<br>N 0%<br>A 1%	L 98%<br>M 2%<br>N 0%<br>A 0%	L 96%<br>M 3%<br>N 0%<br>A 0%	L 97%<br>M 2%<br>N 0%<br>A 0%
Xanthochromia	No	No	No	No	No	No	No	No
Glucose (mg/dL)	73	65	76	78	65	67	72	72
Protein (mg/dL)	**86**	**107**	**68**	**63**	40	**93**	**91**	**116**
JCV RNA					**Positive (mNGS)**	Negative (RT-PCR)		
JCV IgM and neutralizing Abs						Negative		

Bold indicates abnormal result. Abbreviations: A, atypical cells; Abs, antibodies; IgM, immunoglobulin M; JCV, Jamestown Canyon virus; L, lymphocytes; M, monocytes; mNGS, metagenomic next-generation sequencing; N, neutrophils

Appendix Table 2. Key clinical tests performed on cerebrospinal fluid and serum

CSF Test	Result	Date
JC PyV PCR	Negative	4/11/18
HSV 1/2 PCR	Negative	4/17/18
HHV6 PCR	Negative	4/17/18
Whipple PCR	Negative	4/17/18
EBV PCR	Negative	4/25/18
Cryptococcus Ag	Negative	4/17/18
RT-QuIC (CJD)	Negative	4/17/18
Paraneoplastic panel	Negative	4/17/18, 4/20/18, 4/25/18
Cytology	**Increased lymphocytes, occasional reactive cells, no malignant cells**	3/28/18, 4/11/18,4/17/18, 4/20/18, 4/25/18
Flow cytometry	**Predominantly CD5+CD19- T-cells**	3/28/18, 4/11/18,4/17/18, 4/20/18, 4/25/18
IgH rearrangement	Negative	4/11/18, 4/17/18
Amyloid β 42	**Elevated (537.75 pg/mL)**	4/17/18
Total tau	**Elevated (2325.9 pg/mL)**	4/17/18
Phospho tau	Normal (48.4 pg/mL)	4/17/18
Serum Test	Result	Date
JCV PCR	**Positive**	5/18/18
JCV/POWV/LACV IgM	Negative	5/18/18
JCV/LACV NAbs	Negative	5/18/18
HIV 1/2 Ab/Ag	Negative	3/27/18
Treponemal Ab	Negative	3/27/18
Lyme Ab	Negative	3/27/18
T-spot TB	Negative	4/17/18
Cryptococcal Ag	Negative	4/17/18
1–3 Beta D-glucan	Negative	4/17/18
TPO Ab	Negative	4/18/18
Thyroglobulin Ab	Negative	4/18/18
Paraneoplastic panel	Negative	4/20/18
CD19	0% (nl 7%–27%)	4/18/18, 5/23/18, 6/6/18
CD20	0% (nl 3%–20%)	4/18/18, 5/23/18, 6/6/18

Bold indicates abnormal result. Abbreviations: Ab, antibody; Ag, antigen; CJD, Creutzfeldt–Jakob disease; EBV, Epstein-Barr virus; HHV6, human herpesvirus 6; HIV, human immunodeficiency virus; HSV, herpes simplex virus; JC PyV, JC polyomavirus; JCV, Jamestown Canyon virus; LACV, La Crosse virus; Nabs, neutralizing antibodies; POWV, Powassan virus; RT-QuIC, real-time quaking-induced conversion; TPO, thyroid peroxidase

Appendix Table 3. Within-patient SNPs

Small	0.8% of sites with SNPs				33% of SNPs in coding region Nonsynonymous			
Position	CSF	Cortex frozen	Cortex FFPE	Cerebellum frozen	Cerebellum FFPE	HM007356.1	SNP type	Confirmed*
153	G	A	–	A	A	G	Nonsynon#	Y
219	C	T	T	T	–	C	Synonymous	Y
303	T	C	mixed+	T	T	T	Synonymous	Y
336	C	T	T	T	C	C	Synonymous	Y
397	A	G	G	G	G	A	Nonsynon	Y
772	T	C	C	C	–	C	Synonymous	Y
813	G	T	T	–	–	T	Noncoding	Y
814	G	T	T	–	–	G	Noncoding	Y

Medium	0.2% of sites with SNPs				71% of SNPs Nonsynonymous			
Position	CSF	Cortex frozen	Cortex FFPE	Cerebellum frozen	Cerebellum FFPE	HM007356.1	SNP type	Confirmed
180	A	T	T	T	–	T	Stop	N
196	–	mixed	C	T	–	T	Synonymous	Y
303	A	G	–	–	–	A	Nonsynon	N
1201	T	mixed	C	–	–	T	Synonymous	Y
1226	T	C	C	–	–	T	Nonsynon	Y
1227	T	C	C	–	–	T	Nonsynon	Y
1235	T	C	C	–	–	T	Nonsynon	Y
1238	G	A	A	–	–	G	Nonsynon	Y
1291	T	T	T	–	C	T	Synonymous	N
1552	–	A	G	G	–	G	Synonymous	N
1583	–	G	A	A	–	A	Nonsynon	N
2203	T	T	T	C	–	T	Synonymous	N
2232	C	–	–	G	–	C	Nonsynon	N
2415	G	A	–	A	–	A	Nonsynon	Y
3085	–	A	–	A	G	G	Synonymous	N
3498	G	G	G	G	A	G	Nonsynon	N
3502	A	G	G	–	–	A	Nonsynon	Y
3518	C	C	C	C	T	C	Nonsynon	N
3559	C	T	–	–	T	C	Synonymous	N
3918	A	A	–	A	G	A	Nonsynon	N
3946	A	A	A	–	G	A	Nonsynon	N

Large	0.1% of sites with SNPs				27% of SNPS Nonsynonymous			
Position	CSF	Cortex frozen	Cortex FFPE	Cerebellum frozen	Cerebellum FFPE	HM007356.1	SNP type	Confirmed
1891	C	T	T	T	–	T	Synonymous	Y
2346	–	A	A	G	G	G	Nonsynon	Y
2793	T	C	C	–	C	C	Synonymous	Y
3147	A	G	–	G	G	G	Synonymous	Y
3556	C	T	–	–	T	T	Synonymous	Y
4020	T	C	C	C	T	T	Synonymous	Y
4059	T	C	C	T	T	T	Synonymous	Y
5494	–	C	C	T	C	C	Nonsynon	N
5505	–	A	C	A	A	A	Synonymous	N
5713	A	T	T	T	–	T	Nonsynon	Y
5760	A	A	A	A	G	A	Synonymous	N
5850	–	G	G	–	A	G	Synonymous	N
6096	A	G	G	G	G	G	Synonymous	Y
6181	T	C	C	C	–	C	Synonymous	Y
6615	G	A	–	A	–	A	Nonsynon	Y

– indicates insufficient coverage (less than 3 reads at the site of the SNP)
* indicates at least 3 reads in the site of the SNP, and for brain tissue, present in both frozen and FFPE samples
# This mutation resulted in a premature stop codon in the open reading frame for nonstructural protein NSs, which is not essential for virus replication
+ indicates the presence of both C and T variants within the sample

Appendix Table 4. SNPs detected between patient samples and reference JCV sequence from mosquito

Small

Position	HM007356.1	CSF	Cortex frozen	Cortex FFPE	Cerebellum frozen	Cerebellum FFPE	SNP type	Confirmed*
31	A	–	G	–	–	G	Noncoding	N
34	T	–	C	–	–	C	Noncoding	N
400	G	A	A	A	A	A	Nonsynon	Y
401	A	G	G	G	G	G	Nonsynon	Y
529	C	T	T	T	T	T	Synonymous	Y
559	A	G	G	G	G	G	Nonsynon	Y
618	C	T	T	T	T	T	Synonymous	Y
723	G	A	A	A	A	–	Synonymous	Y
784	C	A	A	A	–	–	Noncoding	Y
787	T	G	G	G	–	–	Noncoding	Y
790	T	G	G	G	–	–	Noncoding	Y
849	G	A	A	A	–	–	Noncoding	Y
877	G	A	A	A	–	–	Noncoding	Y
901	C	G	G	–	–	–	Noncoding	Y
904	A	G	G	–	–	–	Noncoding	Y
906	T	A	A	–	–	–	Noncoding	Y
959	G	A	A	–	–	–	Noncoding	Y

Medium

Position	HM007356.1	CSF	Cortex frozen	Cortex FFPE	Cerebellum frozen	Cerebellum FFPE	SNP type	Confirmed*
274	A	–	G	–	–	–	Synonymous	N
290	G	–	A	–	–	–	Nonsynon	N
331	C	T	T	T	–	–	Synonymous	Y
517	C	–	T	T	T	–	Synonymous	Y
577	C	T	T	T	–	–	Synonymous	Y
1025	G	–	A	–	–	–	Nonsynon	N
1053	G	–	A	–	A	–	Nonsynon	N
1186	G	A	A	A	–	–	Synonymous	Y
1219	T	–	C	C	–	–	Synonymous	Y
1429	A	G	G	G	–	G	Synonymous	Y
1496	A	G	G	–	G	G	Nonsynon	Y
1590	C	–	T	T	T	–	Nonsynon	Y
1592	G	–	A	–	–	–	Nonsynon	N
1615	G	T	T	T	T	T	Synonymous	Y
1623	T	C	C	C	C	C	Nonsynon	Y
1627	T	A	A	A	A	A	Synonymous	Y
1656	A	G	G	G	–	G	Nonsynon	Y
1708	G	A	A	–	–	–	Synonymous	Y
1901	A	C	C	C	–	–	Nonsynon	Y
2050	C	T	T	T	T	T	Synonymous	Y
2059	T	–	C	C	C	–	Synonymous	Y
2137	C	T	T	–	T	–	Synonymous	Y
2218	G	A	A	A	A	–	Synonymous	Y
2259	A	–	G	–	–	–	Nonsynon	N
2554	C	T	T	T	–	–	Synonymous	Y
2584	T	C	C	C	–	C	Synonymous	Y
2590	G	A	A	A	–	A	Synonymous	Y
2707	C	–	T	–	–	T	Synonymous	N
2740	G	A	A	A	–	A	Synonymous	Y
2837	T	–	C	C	C	–	Synonymous	Y
3010	T	C	C	–	C	–	Synonymous	Y
3040	C	T	T	–	T	–	Synonymous	Y
3109	T	–	C	–	C	C	Synonymous	Y
3227	T	C	C	C	C	C	Synonymous	Y
3265	C	T	T	T	T	T	Synonymous	Y
3325	T	C	C	C	C	C	Synonymous	Y
3358	C	T	T	T	T	–	Synonymous	Y
3373	T	C	C	C	C	–	Synonymous	Y
3376	T	A	A	A	A	–	Synonymous	Y
3391	G	A	A	A	A	–	Synonymous	Y
3403	T	C	C	C	C	–	Synonymous	Y
3436	T	–	C	C	–	–	Synonymous	Y

Small

Position	HM007356.1	CSF	Cortex frozen	Cortex FFPE	Cerebellum frozen	Cerebellum FFPE	SNP type	Confirmed*
3481	G	–	A	A	–	–	Synonymous	Y
3484	C	–	T	T	–	–	Synonymous	Y
3643	A	G	A	–	A	–	Synonymous	Y
3720	T	C	C	–	–	–	Nonsynon	Y
3757	A	–	G	–	–	–	Synonymous	N
3758	G	–	A	–	–	–	Nonsynon	N
3787	C	–	T	–	–	–	Synonymous	N
3841	T	–	C	–	–	–	Synonymous	N
3959	G	–	A	A	–	A	Nonsynon	Y
3985	T	–	C	C	C	C	Synonymous	Y
4207	A	–	G	–	–	–	Synonymous	N
4285	T	–	C	–	–	–	Synonymous	N
4291	T	–	C	–	–	–	Synonymous	N
4307	A	–	G	–	–	–	Nonsynon	N
4322	T	–	C	–	–	–	Nonsynon	N

Large

Position	HM007356.1	CSF	Cortex frozen	Cortex FFPE	Cerebellum frozen	Cerebellum FFPE	SNP type	Confirmed*
141	C	–	T	–	–	–	Synonymous	N
145	C	–	T	–	–	–	Synonymous	N
177	G	A	A	–	–	–	Synonymous	Y
214	A	G	G	G	G	–	Nonsynon	Y
216	T	C	C	C	C	–	Nonsynon	Y
294	C	T	T	–	T	–	Synonymous	Y
317	T	C	C	–	C	–	Nonsynon	Y
477	C	–	T	–	T	T	Synonymous	Y
684	G	–	A	A	A	–	Synonymous	Y
703	A	–	G	G	G	–	Nonsynon	Y
737	A	G	G	G	G	–	Nonsynon	Y
786	T	–	C	C	C	–	Synonymous	Y
864	T	–	C	C	–	C	Synonymous	Y
894	A	–	G	G	–	G	Synonymous	Y
910	G	–	A	–	A	–	Nonsynon	N
957	T	–	C	–	–	C	Synonymous	N
962	A	–	A	–	–	–	Nonsynon	N
987	T	C	C	–	C	–	Synonymous	Y
997	T	C	C	C	C	C	Nonsynon	Y
1067	G	A	A	A	A	A	Nonsynon	Y
1158	G	A	–	A	–	–	Synonymous	Y
1176	A	G	G	G	–	–	Synonymous	Y
1200	A	G	G	G	–	–	Synonymous	Y
1317	C	T	T	–	T	–	Synonymous	Y
1366	T	C	C	–	C	–	Synonymous	Y
1509	A	–	G	–	G	–	Synonymous	N
1609	A	G	G	G	–	G	Nonsynon	Y
1665	A	C	C	–	C	–	Synonymous	Y
1744	G	A	A	–	–	–	Nonsynon	Y
1761	G	–	A	–	A	–	Synonymous	N
1779	C	–	T	–	T	–	Synonymous	N
2040	T	–	C	C	C	C	Synonymous	Y
2112	C	T	T	–	–	T	Synonymous	Y
2151	C	–	T	–	–	–	Synonymous	N
2152	A	–	G	–	–	–	Nonsynon	N
2165	A	–	A	–	–	A	Nonsynon	N
2343	T	–	C	C	C	C	Synonymous	Y
2349	G	A	A	A	A	–	Synonymous	Y
2353	C	T	T	T	T	–	Synonymous	Y
2409	C	T	T	T	T	–	Synonymous	Y
2439	C	T	T	T	T	–	Synonymous	Y
2604	T	–	C	–	C	–	Synonymous	N
2626	T	–	T	–	T	–	Nonsynon	N
2730	C	T	T	–	T	T	Synonymous	Y
2826	G	–	A	A	A	–	Synonymous	Y
2872	G	A	A	A	A	–	Nonsynon	Y
2874	C	–	T	T	T	–	Nonsynon	Y

Small Position	HM007356.1	CSF	Cortex frozen	Cortex FFPE	Cerebellum frozen	Cerebellum FFPE	SNP type	Confirmed*
2883	T	–	C	C	C	C	Synonymous	Y
2976	C	T	–	–	T	T	Synonymous	Y
2985	G	A	A	–	A	A	Synonymous	Y
3000	C	–	T	T	–	T	Synonymous	Y
3012	C	T	T	T	–	T	Synonymous	Y
3145	T	C	C	–	C	C	Synonymous	Y
3168	G	A	A	–	A	A	Synonymous	Y
3180	A	G	G	–	G	–	Synonymous	Y
3255	C	–	T	T	T	T	Synonymous	Y
3333	C	T	T	T	–	–	Synonymous	Y
3413	C	T	T	T	T	–	Nonsynon	Y
3597	G	A	A	A	A	–	Synonymous	Y
3606	A	G	G	–	G	G	Synonymous	Y
3673	G	A	A	A	–	–	Nonsynon	Y
3810	T	–	C	–	C	C	Synonymous	Y
3846	T	C	C	–	C	–	Synonymous	Y
3891	G	–	A	A	A	–	Synonymous	Y
3903	T	–	G	G	G	G	Synonymous	Y
3918	C	T	T	T	T	T	Synonymous	Y
4080	A	G	G	G	–	–	Synonymous	Y
4146	A	–	G	G	–	G	Synonymous	Y
4203	G	–	A	A	A	–	Synonymous	Y
4368	C	T	T	T	T	–	Synonymous	Y
4515	T	–	C	–	C	–	Synonymous	N
4626	T	–	C	–	C	–	Synonymous	N
4668	G	–	–	–	A	–	Synonymous	N
4680	C	–	T	–	T	–	Synonymous	N
4695	G	–	A	–	A	–	Synonymous	N
4773	A	–	G	–	G	–	Synonymous	N
4776	C	–	T	–	T	–	Synonymous	N
4827	C	–	T	–	T	T	Synonymous	Y
5022	T	C	C	C	C	C	Synonymous	Y
5053	G	A	A	A	A	A	Nonsynon	Y
5280	G	–	A	A	–	A	Synonymous	Y
5529	G	–	A	A	A	A	Synonymous	Y
5539	C	–	T	T	–	T	Synonymous	Y
5760	A	A	A	A	A	G	Synonymous	N
5823	G	A	A	A	–	A	Synonymous	Y
5874	C	–	T	–	T	T	Synonymous	Y
5991	G	A	A	A	A	A	Synonymous	Y
6090	A	G	G	G	G	G	Synonymous	Y
6183	G	A	A	A	A	–	Synonymous	Y
6210	A	–	G	G	G	G	Synonymous	Y
6277	T	C	C	–	–	C	Synonymous	Y
6315	A	–	G	G	G	G	Synonymous	Y
6519	C	T	T	T	T	T	Synonymous	Y
6615	A	G	A	–	A	–	Nonsynon	Y
6633	C	–	T	–	T	–	Synonymous	N
6648	A	–	G	–	G	–	Synonymous	N
6750	G	–	A	–	A	–	Synonymous	N
6822	G	–	A	–	–	–	Synonymous	N
6834	T	–	C	–	–	–	Synonymous	N
6864	T	–	C	–	C	–	Noncoding	N

–indicates insufficient coverage (less than 3 reads at the site of the SNP)

* indicates at least 3 reads in the site of the SNP, and for brain tissue, present in both frozen and FFPE samples

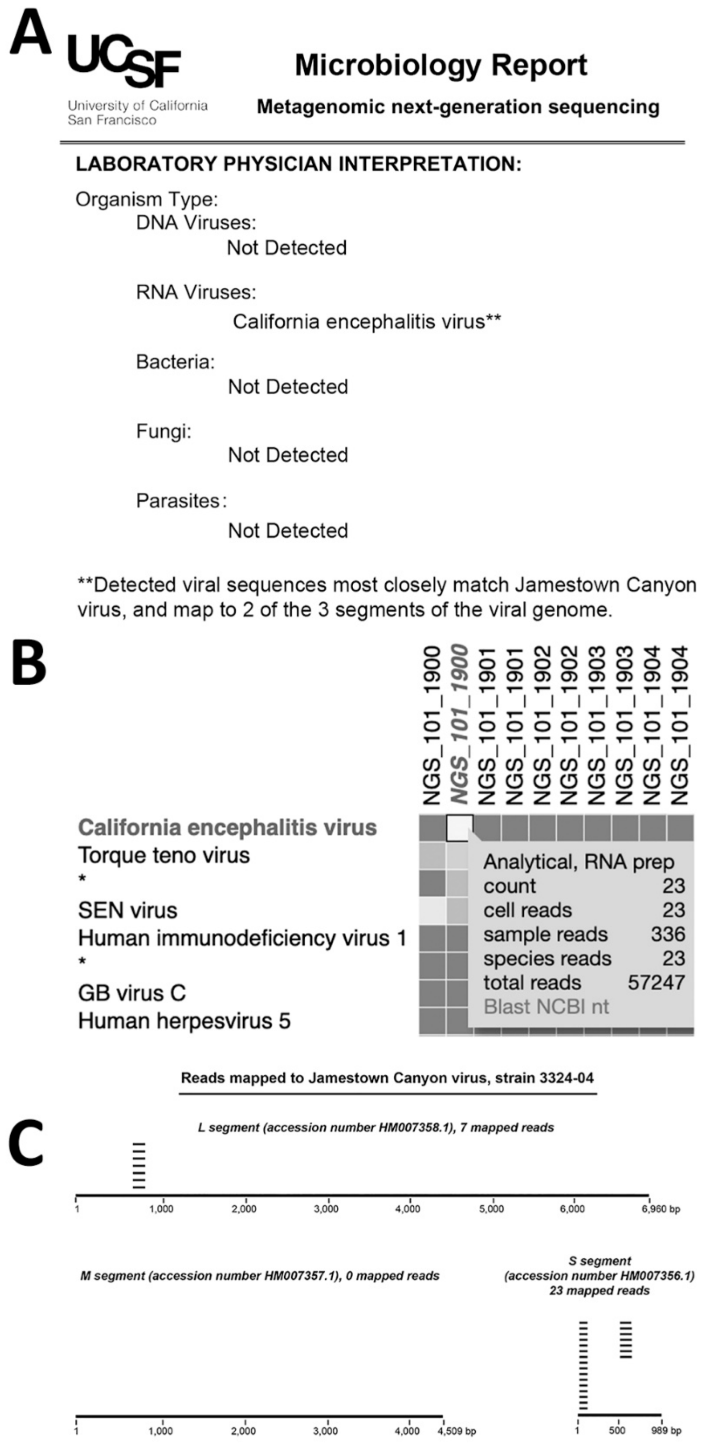

Appendix Figure 1. Clinical metagenomic next-generation sequencing of cerebrospinal fluid. (A) Clinical mNGS results report, showing detection of California encephalitis virus (the viral genome in NCBI GenBank that most closely matched Jamestown Canyon orthobunyavirus). Reads were identified from the S and L but not M segments in this initial sequencing run. (B) Heat map of aligned sequence reads corresponding to detected pathogens. Each column is a patient CSF sample, while each row is a

detected viral species. The asterisks denote taxonomic categories classified above the species level (e.g., genus, family, etc.). The pop-up window corresponding to the highlighted cell displays the total reads and species reads for California encephalitis virus. (C) Coverage maps for the Jamestown Canyon virus reads identified by mNGS. The number of reads mapping to the viral genome are 7, 0, and 23 to the L, M, and S segments, respectively.

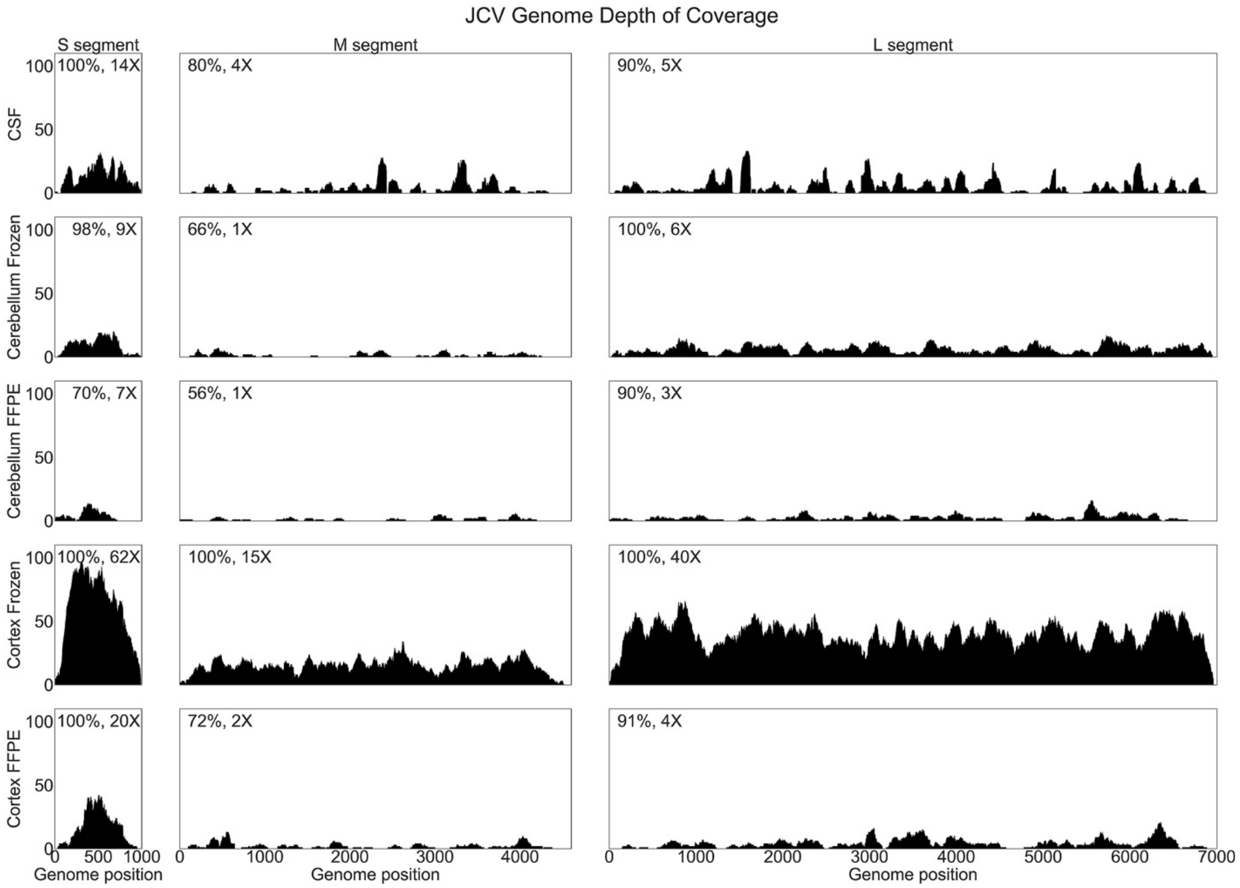

Appendix Figure 2. JCV genome coverage plots. Depth of coverage, based on unique JCV reads, for each sample and preparation type. For each segment, the percent coverage and mean depth are indicated at top left.
